# In Vitro Prediction of Skin-Sensitizing Potency Using the GARDskin Dose–Response Assay: A Simple Regression Approach

**DOI:** 10.3390/toxics12090626

**Published:** 2024-08-24

**Authors:** Robin Gradin, Fleur Tourneix, Ulrika Mattson, Johan Andersson, Frédéric Amaral, Andy Forreryd, Nathalie Alépée, Henrik Johansson

**Affiliations:** 1 Senzagen AB, 22381 Lund, Sweden; ulrika.mattson@senzagen.com (U.M.); johan.andersson@senzagen.com (J.A.); andy.forreryd@senzagen.com (A.F.); henrik.johansson@senzagen.com (H.J.); 2 L’Oréal, Research & Innovation, 93600 Aulnay-sous-Bois, France; fleur.tourneix@loreal.com (F.T.); frederic.amaral@loreal.com (F.A.); nathalie.alepee@loreal.com (N.A.)

**Keywords:** NAM, GARDskin Dose–Response, sensitizing potency, quantitative risk assessment, point of departure

## Abstract

Toxicological assessments of skin sensitizers have progressed towards a higher reliance on non-animal methods. Current technological trends aim to extend the utility of non-animal methods to accurately characterize skin-sensitizing potency. The GARDskin Dose–Response assay has previously been described; it was shown that its main readout, cDV_0_ concentration, is associated with skin-sensitizing potency. The ability to predict potency from cDV_0_ in the form of NESILs derived from LLNAs or human NOELs was evaluated. The assessment of a dataset of 30 chemicals showed that the cDV_0_ values still correlated strongly and significantly with both LLNA EC3 and human NOEL values (ρ = 0.645–0.787 [*p* < 1 × 10^−3^]). A composite potency value that combined LLNA and human potency data was defined, which aided the performance of the proposed model for the prediction of NESILs. The potency model accurately predicted sensitizing potency, with cross-validation errors of 2.75 and 3.22 fold changes compared with NESILs from LLNAs and humans, respectively. In conclusion, the results suggest that the GARDskin Dose–Response assay may be used to derive an accurate quantitative continuous potency estimate of skin sensitizers.

## 1. Introduction

Chemicals possess a wide range of physiochemical properties. While many are beneficial, making them ubiquitous in industries and society, some can induce hazardous effects in humans. Therefore, it is essential to investigate the potential of chemicals to induce adverse effects. One type of hazard that is routinely screened for is the capacity to induce skin sensitization [1,2]. Skin sensitization is a hypersensitivity reaction that follows a topical exposure to an inducing chemical, leading to the establishment of a specific immunological memory [3,4]. The underlying mechanisms of this reaction have been extensively studied and explained, and the major key events (KEs) are recognized and have been summarized in an Adverse Outcome Pathway (AOP) [5]. As the sensitization reaction results in an immunological memory, a sensitized individual will suffer from adverse effects upon repeated exposure to that specific chemical, making the initial avoidance of hazardous exposure levels imperative.

The induction of skin sensitization is a dose-dependent event, and the likelihood that sensitization occurs typically increases with dose [6]. However, chemicals’ efficiencies to induce sensitization vary greatly [7]. For example, a strong skin sensitizer may cause sensitization from a few micrograms of topical exposure, while weak sensitizers would require milligrams to reach a similar probability of sensitization on the same surface area. Therefore, it is not only relevant to evaluate the ability of a chemical to induce skin sensitization, but also to characterize an identified skin sensitizer’s potency, i.e., to derive an estimate of an exposure level that is not expected to induce skin sensitization in the general population.

Traditionally, the assessment of skin sensitizers has relied on information from in vivo assays. Guinea pig models were among the earliest standardized methods, which included the Buehler assay [8,9,10] and the Guinea Pig Maximization Test (GPMT) [11]. These were largely superseded by the more recent murine Local Lymph Node Assay (LLNA) [12,13,14], which brought several improvements over the guinea pig assays. These include aspects of animal welfare and an improved scientific rationale, as the assay provides an objective and quantitative readout [15]. Further, while potency information could be gathered from the guinea pig models [6], the LLNA incorporates dose–response measurements, and the derived effective concentration for a stimulation index of 3 (EC3) serves as an immediate indicator of sensitizing potency, which has been shown to correspond well with human-derived potency data [16,17,18,19]. Human potency data are often described in terms of No Observed Effect Levels (NOELs) or Lowest Observed Effect Levels (LOELs) from Human Repeat Insult Patch Tests (HRIPTs). However, testing on humans is problematic [20], especially given the potential severity and enduring effects of skin sensitization, and is typically avoided unless used in confirmatory scenarios where a lack of response has been demonstrated [21]. Nevertheless, when available, data from both humans and LLNAs may be used as information sources to derive No Expected Sensitization Induction Levels (NESILs), which may be used as a Point of Departure (PoD) in risk assessment strategies [22,23].

More recent advancements in the field of skin-sensitization assessment, however, are being made toward the development of non-animal methods, or New Approach Methodologies (NAMs), aiming to replace animal tests, not only for hazard identification but also for hazard characterization and potency evaluation. An initial milestone, which has to a significant degree already been realized, is the establishment of NAMs capable of accurately and reliably identifying skin-sensitizing hazards. Today, many different NAMs can be used to generate such hazard information, and validated and regulatory approved methods are described in various OECD test guidelines [24,25,26], each of which is associated with a specific KE of the AOP. However, none of the methods described in the TGs have so far been approved for stand-alone use. Therefore, endeavors have also been made to integrate results across NAMs to increase confidence in their joint classification outcomes, using so-called Defined Approaches for Skin Sensitization (DASS) [27,28,29]. In addition to these NAMs, skin-sensitizing hazard data can also be obtained using in silico methods [30,31,32,33], which may contribute to weight-of-evidence assessments.

A second milestone in the replacement of animal tests is the development of methods capable of informing about chemicals’ skin-sensitizing potencies. Indeed, some potency information may already be derived in a discrete manner, as described in the current OECD test guideline 497, where integrated testing strategies incorporating multiple information sources (including in chemico, in vitro, and in silico) are used to predict sensitizing potency per the globally harmonized system for classification and labelling (UN GHS) [29]. However, the apogee of NAM development for the purpose of potency assessment is, nevertheless, likely a readout provided as a quantitative continuous potency value. Some progress has already been made towards this objective. For example, probabilistic approaches based on Bayesian or Artificial Neuronal Network (ANN) frameworks that incorporate multiple information sources, including several NAMs, have been described for the prediction of PoDs [34,35,36,37]. Similarly, several alternative regression models based on the output of established NAMs have been proposed for the prediction of LLNA EC3 values [38]. Besides methods incorporating data from individual NAMs, novel test methods have also been proposed for directly generating potency information [24,39].

While the development of NAMs for the assessment of skin sensitizers progresses, frameworks for interpreting their results and managing risks are also being established. For example, Next-Generation Risk Assessment (NGRA) strategies that incorporate information from several NAMs have recently been proposed [40,41].

We have previously described an extended protocol of the GARDskin assay that incorporates dose–response measurements to derive a concentration estimate that correlates strongly and significantly with both LLNA EC3 and human NOELs [42]. The classical GARDskin assay is an OECD-approved assay described in test guideline 442E [26], together with other methods addressing the key event of dendritic cell activation. The GARDskin assay, and the dose–response adaptation, relies on the monitoring of genetic changes in a biomarker signature following chemical exposure. The biomarker signature includes several genes relevant to immune activation, including cd86, hmox1, nqo1, and nlrp12 [43]. The dose–response adaptation studies the summarized gene response over multiple concentrations to identify the lowest concentration capable of inducing a positive classification in the GARDskin assay. As such, the main methodology is a typical toxicological approach, which is also applied in the LLNA, where a PoD is determined from a dose–response curve and used as a measure of potency.

In this work, we further characterize the GARDskin Dose–Response assay and provide results that show that its readout, the cDV_0_ value, correlates significantly with established potency metrics on a larger dataset and can be used to accurately predict potency. Notably, the redundancy and potential ambiguity of having repeated NESILs derived from the LLNA and human NOELs for model fitting was rectified using a composite potency value describing a latent potency signal. This approach improved the predictive performance compared with methods relying on individual references, generating NESIL predictions relevant for risk assessment frameworks such as NGRA [40,41].

## 2. Materials and Methods

### 2.1. The GARDskin Dose–Response Assay

The GARDskin Dose–Response assay is based on the protocol of the GARDskin assay, publicly available in the Tracking System for Alternative methods towards Regulatory acceptance (TSAR) [44]. The assay measures the expression levels of genes in the GARDskin Genomic Prediction Signature (GPS) following treatment with a test chemical to the cell system [26,43,45], i.e., the Senzacell cell line (ATCC depository PTA-123875). The interpretation of gene response is made with a support vector machine (SVM) (explicitly described in supporting documents to the OECD TG 442E [46]), which outputs a decision value (DV) on which the subsequent classification is based. A test chemical is classified as a skin sensitizer if the generated mean DV is greater than zero (DV ≥ 0) and as a non-sensitizer if the mean DV is less than zero (DV < 0). 

The GARDskin Dose–Response assay extends the standard protocol by evaluating the test chemical at several concentrations [42]. DVs are calculated for every concentration and the lowest concentration expected to induce a positive classification is determined, which corresponds to the lowest concentration that predicts a DV of 0. Practically, this concentration can be estimated using linear interpolation between the two closest concentrations that generate predictions on the opposite sides of the GARDskin classification threshold. The estimated concentration is the main readout of the GARDskin Dose–Response assay, termed cDV_0_ [42].

In more detail, a test item assessment starts with an examination of the cytotoxic properties of the chemical, using a protocol based on propidium iodide staining and flow cytometry analysis. A concentration inducing low-to-non-toxic conditions, as determined by the relative viability of the cell system following exposure, is determined. This concentration is termed the GARD input concentration and constitutes the sole assessment concentration in the standard GARDskin assay and the highest evaluated concentration in the GARDskin Dose–Response assay. Subsequent concentrations in the dose–response assay are typically calculated using a dilution series with a dilution factor of approximately 0.5. The actual design of the concentration curve can be modified for particular purposes, i.e., a higher number of replicates with smaller steps between concentrations may result in reduced uncertainty of an estimated cDV_0_ value but at the cost of resources. As this study comprises chemicals from several separate experiments, the designs of the concentration curves are not identical. For example, in Gradin et al.’s study [42], chemicals were analyzed by evaluating 12 different concentrations with a dilution factor of 0.6 and a single replicate per concentration. Since then, data have been acquired using more standardized dilution schemes optimized from accumulated observations, typically including 6 concentrations with two or three replicates. The actual dilution schemes used for each chemical in this study are available in Supplementary Tables S2 and S3.

Cells are treated with the test chemical at the determined concentrations for 24 h, following which cells are harvested and total RNA is isolated and purified. Gene expression levels are quantified using the NanoString nCounter system using a custom GARDskin codeset, as described previously [26,45]. The data analysis pipeline for transforming raw gene expression levels into decision values has been publicly described, including all parameter values [46]. For the dose–response analysis, the decision values are examined against the treatment concentrations to verify the presence of a dose–response relationship. A cDV_0_ concentration is estimated when a dose-dependent response is observed, using linear interpolation between the two closest points (in terms of concentration) that are on adjacent sides of the classification threshold.

### 2.2. Dataset

This work was based on a dataset of 30 chemicals, as listed in Table 1. More complete details regarding the chemicals are presented in Supplementary Table S1. GARDskin Dose–Response data for 18 of the chemicals were previously described in Gradin et al.’s work [42]. In that work, chemicals were selected to span a wide range of expected potency values while preferably having both human and LLNA reference data. In addition, they were selected to cover the potency span relatively evenly to allow for an efficient correlation analysis between cDV_0_ and potency references. That dataset of 18 chemicals was extended here with 12 new chemicals. The new chemicals raised skin-sensitization alerts and had LLNA and human NOEL data. The chemicals were butyl resorcinol (18979-61-8); citral (5392-40-5); chlorpromazine (50-53-3); (R)-(+)-Limonene (5989-27-5); alpha-iso-Methylionone (127-51-5); phenylacetaldehyde (122-78-1); ethyl acrylate (140-88-5); cinnamic alcohol (104-54-1); 3-Propylidenephthalide (17369-59-4); 5-Methyl-2,3-hexanedione (13706-86-0); and carvone (6485-40-1). A majority of weak and moderate, i.e., UN GHS Cat. 1B, skin sensitizers were selected to reduce the uncertainty in the estimated relationship between cDV_0_ and the sensitization potency for this category of ingredients, as precision in this potency range may be of particular relevance for the cosmetics industry.

The analysis included repeated runs for 10 of the chemicals. Repeated cDV_0_ values were merged by their geometric mean values before incorporation in downstream calculations (including correlation evaluations and regression analyses). Individual cDV_0_ values and repeated runs’ geometric means are described in Table 1 and in Supplementary Table S1. The geometric mean was used as it consistently maintains the relative differences between measurements. For example, the geometric mean of 0.5 μg/mL and 2 μg/mL is 1 μg/mL, which is 2-fold from either of the original values.

LLNA EC3, human NOELs, and human Lowest Observed Effect Levels (LOELs) were collected from the published literature [47,48,49,50,51,52,53] (see Supplementary Table S1 for details). LLNA EC3 values were converted into NESIL estimates in μg/cm^2^ by multiplying the percentage values by 250 [54].

### 2.3. Creation of a Composite Potency Value

The reference composite potency value was created by fitting a robust errors-in-variables model between NESIL values derived from LLNA EC3 and human NOELs, and by orthogonally projecting the datapoints onto the fitted line, creating the composite potency values. More explicitly, a Passing–Bablok regression model [55] was fitted between the log-transformed NESILs from LLNA EC3 and human NOEL values (see Table 1 for a description of the data). The 25 chemicals with continuous LLNA and human data were included in the fit. The Passing–Bablok fit was created in R (version 4.2.0) [56] with the R-package Deming (version 1.4) [57]. Confidence intervals for the fitted coefficients were estimated using bootstrap [58], taking 10,000 bootstrap replicates. The composite potency values were calculated as follows.

Given the linear PB fit as line *l* in the form *y = ax + b*, and data point *p* with coordinates (*x_i_*, *y_i_*), the coordinates of the projection of point *p* onto *l* was calculated by finding the intersect between line *l* and the line that is orthogonal to *l* and passes through coordinates (*x_i_*, *y_i_*), as described by Equation (1). The point’s projected coordinates (${\hat{x}}_{i}$, ${\hat{y}}_{i}$) are thus given by Equations (2) and (3).

(1) $$y=-\frac{x}{a}+y_{i}+\frac{x_{i}}{a},$$

(2) $${\hat{x}}_{i}=\frac{y_{i}+\frac{x_{i}}{a}+b}{a+\frac{1}{a}},$$

(3) $${\hat{y}}_{i}=a\times {\hat{x}}_{i}+b,$$

The relative distances between the projected points were calculated with the Pythagorean theorem, using the data point with the smallest composite potency value as a reference (note that $\min\left(\hat{x}\right)$ and $\min\left(\hat{y}\right)$ refer to the same data point). As the relative distances between the projected points may be inflated in this step, the data were scaled by the expected inflation rate per original potency unit. The relative distances were calculated as described in Equation (4).

(4) $$d_{i}=\sqrt{{\left({\hat{x}}_{i}-\min\left(\hat{x}\right)\right)}^{2}+{\left({\hat{y}}_{i}-\min\left(\hat{y}\right)\right)}^{2}}\times \frac{1}{\sqrt{1+a^{2}}},$$

where *d_i_* is the distance between chemical *i* and the chemical with the smallest composite potency value on the composite potency scale, ${\hat{x}}_{i}$ is the projected x-coordinate for chemical *i*, ${\hat{y}}_{i}$ is the projected y-coordinate for chemical *i*, $\min\left(\hat{x}\right)$ and $\min\left(\hat{y}\right)$ correspond to the minimum values of the projected x- and y-coordinates for the set of chemicals, and *a* is the slope of the fitted PB model onto which the projections are made. Finally, the location of the composite potency value was shifted so that the average differences between the composite scale and the projected x- and y-coordinates were minimized (see Equation (5)).

(5) $$cPV=d+\frac{Avg\left(\hat{x}-d\right)+Avg\left(\hat{y}-d\right)}{2},$$

where *cPV* is the composite potency value, *d* is the vector of distance calculated in Equation (4), $\hat{x}$ and $\hat{y}$ are the projected x- and y-coordinates, and *Avg* is a function for the calculation of the arithmetic mean.

### 2.4. Fitting of Potency Prediction Models

Two main types of models were considered for predicting potency values from cDV_0_. The first one was standard linear regression, where overall prediction errors are minimized. The other type of model was a robust implementation of linear regression, where the fit was achieved using iterative re-weighted least squares, incorporating Huber loss to down-weight samples with relatively outlying values [59]. The constant *k* in the Huber weighting was kept at the default value of 1.345. The standard regression models were fitted using R (version 4.2.0) and base package stats (version 4.2.0) [56]. The robust regression models were fitted in R (version 4.2.0) with the package MASS (version 7.3-56) [60].

The performances of the fits were evaluated using repeated cross-validation, with 50 repeats and 10 folds. Classification accuracy was based on the absolute geometric mean fold change, defined as described in Equation (6). The cross-validation performance was summarized within repeats and then across the repeats.

(6) $$Absolute\;geometric\;mean\;fold\;change=e^{\frac{\sum_{i=1}^{n}\left|\log\left(\frac{{Prediction}_{i}}{{Reference}_{i}}\right)\right|}{n}}$$

where *Prediction_i_* is the potency prediction of chemical *i*, *Reference_i_* is the reference potency value for chemical *i*, and *n* is the number of chemicals. Note that *Prediction_i_* and *Reference_i_* are not log-transformed in Equation (6).

### 2.5. General Statistical Calculations and Visualizations

All calculations were performed in R (version 4.2.0) [56]. Correlation metrics and regression models were calculated and fitted on log10-transformed concentrations or dose values, unless explicitly stated otherwise. Linear correlations were calculated with the Pearson correlation coefficient and rank correlations with the Spearman correlation coefficient. Calculations of prediction errors were based on relative differences, expressed as fold changes. Aggregated performance figures summarized over a set of chemicals were calculated as absolute geometric mean fold changes (see Equation (6)).

**Figure 1:**
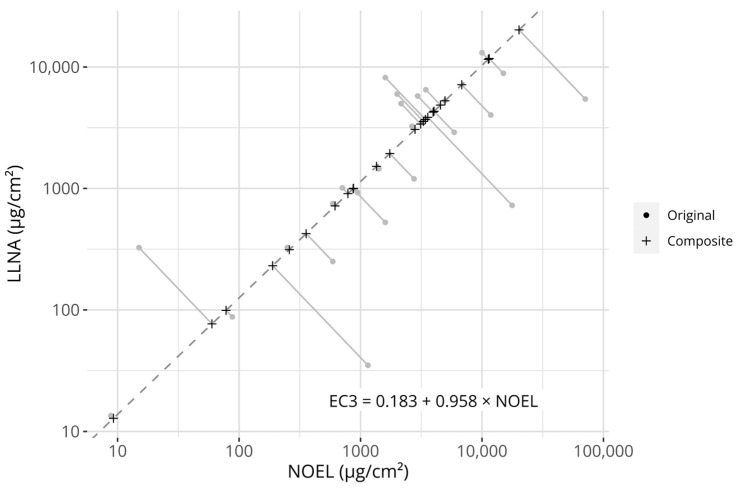
Visualization of the creation of the composite potency values. The grey circular points describe the original reference values (i.e., LLNA EC3 values and human NOEL values). The dashed line describes the Passing–Bablok regression model fitted to the potency references (its equation is described in the figure). Plus signs describe the projection of the original reference values onto the model. The grey linear segments visualize the projections. Axes are log-transformed.

**Figure 2:**
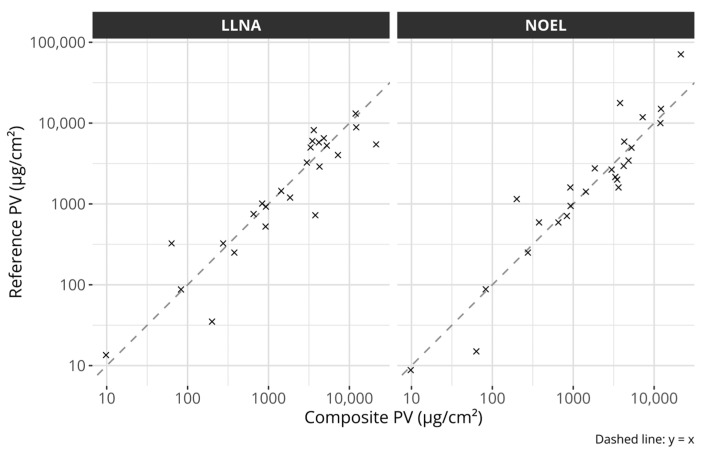
Scatter plots comparing the composite potency values with the individual references. The left plot compares the composite potency values with LLNA EC3 values and the right plot with human NOEL values. The dashed lines describe the identity lines, i.e., y = x. All axes are log-transformed.

**Figure 3:**
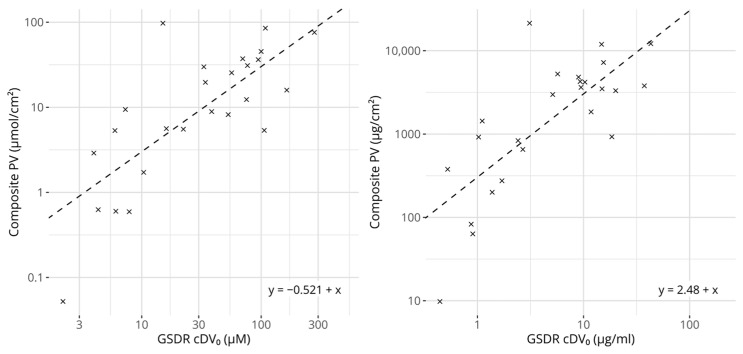
Scatter plots comparing GARDskin Dose–Response cDV_0_ values with composite potency values. Concentration units are expressed in molar-based concentrations on the left and mass-based concentrations on the right. The dashed lines describe the regression models for predicting potency from cDV_0_ values. The equations of the lines are described in the figures. All axes are log-transformed.

Figure 1, Figure 2 and Figure 3 were created using ggplot2 (version 3.4.0) [61].

## 3. Results

### 3.1. Potency Information in cDV_0_ Values

The GARDskin Dose–Response assay is a method for the quantitative assessment of skin-sensitizing potency. cDV_0_ has been shown to be significantly associated with skin-sensitizing potency, defined from either LLNA EC3 values or human NOEL values. To provide a more detailed understanding of these associations, and to examine methods capable of predicting such potency values from cDV_0_, data on 12 chemicals were generated and added to a dataset of 18 previously described chemicals [42]. The results for the complete dataset of 30 chemicals are described in Table 1.

The correlations between cDV_0_ values and LLNA EC3 and human NOELs were first assessed (see Table 2). As can be seen, both linear and rank correlations are significant and range from 0.645 to 0.787. Generally, the cDV_0_ values appear more strongly correlated with LLNA EC3 than with human NOELs. Moreover, the magnitudes of the correlation scores are similar to those calculated between the two references.

### 3.2. Creation of Reference Composite Potency Values

As shown, the cDV_0_ values correlate significantly with both LLNA EC3 and human NOELs, which suggests that the ranking of chemicals obtained from the GARDskin Dose–Response assay is, in itself, informative of potency. However, for the metric to be useful in NGRA, the cDV_0_ value is advantageously translated into a more appropriate unit such as the more readily interpretable μg/cm^2^.

However, two distinct sets of references are available for constructing a potential prediction model, i.e., NESILs derived from either LLNA EC3 values or human NOEL values. While it may be argued that the human endpoint is the more relevant one (as we are attempting to predict sensitization in humans), LLNA EC3 values are a more consistent estimator of a PoD, as human NOELs do not necessarily represent the highest possible non-sensitizing concentration (only the highest observed non-sensitizing concentration). As both metrices have potential advantages and disadvantages, while simultaneously describing an underlying potency signal, it was hypothesized that one could leverage both information sources against each other to create a composite potency value to which a model could be fitted. Therefore, a robust errors-in-variables model, a Passing–Bablok (PB) regression model, was fitted between the 25 chemicals with NESILs from both LLNA EC3 and human NOEL values. The fit between the measures is displayed in Figure 1. The fitted coefficients of the PB model are described in Table 3. The estimated coefficients were close to, and not significantly different from, the identity line (slope = 1, intercept = 0), indicating that no systematic differences between the references could be detected on the examined dataset.

The composite potency values were created by projecting the individual data points onto the fitted line. The individual reference values and the composite scores are described in Table 1. Figure 2 compares the composite scores with the original potency references. The figure suggests that the composite values capture the overall potency information from either reference well.

### 3.3. Prediction of Potency Values from cDV_0_

Figure 3 shows a scatter plot comparing the cDV_0_ values with the derived composite potency values. As expected, based on the underlying reference values, they correlate strongly and significantly. The linear correlation for molar-based concentrations was estimated to be 0.770 (*p* = 6.68 × 10^−6^) and the rank correlation to be 0.710 (*p* = 1.07 × 10^−4^). The corresponding values for the mass-based concentrations were 0.762 (*p* = 9.56 × 10^−6^) and 0.709 (*p* = 1.10 × 10^−4^), for linear and rank correlation respectively. Interestingly, for the 25 chemicals that had references from LLNA EC3 and human NOEL values, the correlation scores between the composite potency values and cDV_0_ were higher compared with any correlation score calculated between cDV_0_ and either of the individual references.

Regression models were fitted with the aim of predicting the composite potency values from the cDV_0_ values. Two main variants of regression models were considered: a standard linear regression model and a robust regression model that reduced the influence of deviating observations. The model types were fitted both with and without a parameter for a slope. When the slope was not estimated, a constant value of 1 was assumed. The performances of all four types of models were examined using repeated cross-validation, which showed that the prediction errors were consistently smaller for the models that only estimated an intercept. The two regression techniques produced relatively similar results, but the smallest errors were observed for the robust regression. Therefore, the robust regression model that only included an intercept was selected as the most appropriate for potency predictions. From the cross-validation, the prediction errors for both molar- and mass-based models were estimated at 2.75 and 3.22 fold changes compared with the NESIL values from the LLNA EC3 and human NOELs, respectively.

For comparative reasons, and to allow for the assessment of the utility of the composite potency values for the fitting of the models, the cross-validation procedure was repeated using the individual potency references for fitting and evaluating the regression models (e.g., a model was trained and evaluated on LLNA data). The result from this cross-validation is described in Table 4. As can be seen, the prediction errors of these models were generally higher compared with the errors obtained for the models fitted to the composite potency values, supporting the hypothesis that it may be beneficial to leverage potency references against each other.

Based on available data and the presented results, the robust regression model that only estimated an intercept and that was fitted on the composite potency values was found to be the overall best-performing potency prediction model. Table 5 shows the fitted coefficients. Given the simplicity of the final models and the fact that they were fitted on log-transformed values, predictions can in practice be obtained by multiplying a cDV_0_ value by a constant, which equals the value of the intercept expressed in base 10 (see Equations (7) and (8); coefficients are rounded to three significant figures).

Predicted potency in μmol/cm^2^ = cDV_0_ in μM × 0.301, (7)

Predicted potency in μg/cm^2^ = cDV_0_ in μg/mL × 304, (8)

The fitted values of the proposed models and their predictions for the samples not included in the training set (i.e., the five chemicals without complete references; data are detailed in Supplementary Table S1) were further examined. The geometric mean prediction errors expressed in fold change were estimated to be 2.69 and 3.12 compared with NESILs from LLNA EC3 and human NOELs, respectively (for both mass- and molar-based concentrations). For comparison, the prediction error of LLNA EC3 compared with human NOELs was 2.63 fold changes. On the same set of 25 chemicals, the GARDskin Dose–Response’s prediction error of human NOELs was 2.94 fold changes. While the LLNA predicted human potency with an overall lower error on this set of chemicals, the difference in performance compared with the proposed prediction model was not significant (*p* = 0.508 [Wilcoxon signed-rank test]). 

**Figure 4:**
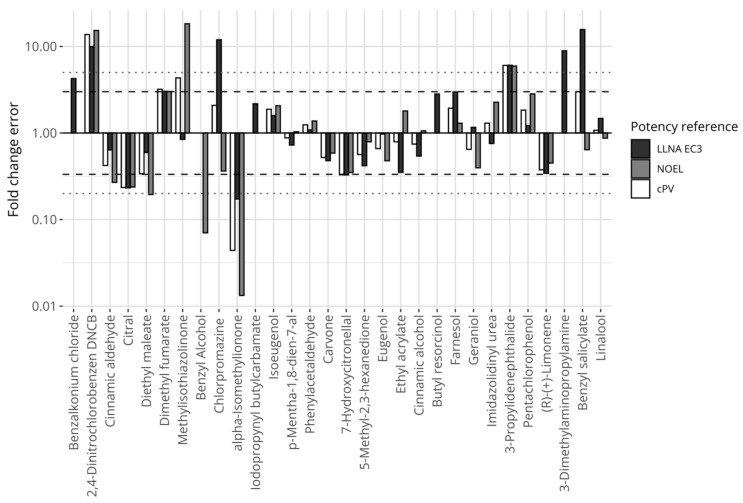
Prediction errors expressed in fold changes for the model defined on mass-based concentrations, comparing predictions of the proposed potency model with reference values. The *y*-axis is log-transformed. The dashed horizontal lines mark the 3-fold thresholds and the dotted horizontal lines describe the 5-fold thresholds.

Figure 4 displays the relative errors between the proposed model’s predictions and the individual references’ potency values for the chemicals, including LLNA EC3 (black bars), human NOELs (grey bars), and the composite reference values (white bars). Fourteen chemicals were consistently predicted within 3 fold changes compared with any of the references (i.e., the largest fold change for any reference was below 3). Three chemicals were predicted with a greater than 5-fold difference compared with any of the references (i.e., the smallest fold change for any reference was greater than 5). These chemicals were 2,4-dinitrochlorobenzene and 3-propylenephthalide (underpredicted), and alpha-isomethylionone (overpredicted). 

Finally, considering the comparison between potency predictions and the reference values, due to the simplicity of the proposed potency prediction model, the correlation coefficients between predictions and references are identical to those described in Table 2 (ranging between 0.645 and 0.787 when compared with LLNA EC3 and human NOELs).

## 4. Discussion

The development of NAMs able to inform on chemicals’ skin-sensitizing hazards has progressed relatively rapidly and far, and several methods have gained regulatory approval for identifying hazards, following the demonstration of their reliabilities and performances [24,25,26,29]. In addition, more recent advancements have seen integrated testing strategies approved for categorical potency assessment in line with the subcategorization defined by the UN GHS [29]. While all of these progressions reduce and replace the necessity of animal methods for the assessment of skin sensitizers, there are still areas where NAMs can be further improved. One such area is the assessment of skin-sensitizing potency, particularly considering the derivation of quantitative continuous potency metrices that may act as replacements for, e.g., the well-established LLNA EC3 value. This is an active field of research and several interesting methods have been described [34,36,37,38,39].

In this work, we have expanded on previously published results, describing how the GARDskin assay can be used to derive a continuous concentration metric, the cDV_0_ value, associated with skin-sensitizing potency. Specifically, the GARDskin Dose–Response assay extends the validated GARDskin assay’s protocol by incorporating measurements at multiple concentrations. Using the derived dose–response data, the lowest concentration capable of inducing a positive classification is identified. Here, we describe such cDV_0_ concentrations for a set of 30 chemicals and further characterize their association with both human and LLNA data. It was confirmed that cDV_0_ values maintained strong and significant correlations with both LLNA EC3 and human NOELs on this extended set of chemicals. Generally, the correlation coefficients were higher for LLNA EC3 compared to human NOELs. This could potentially be attributed to the fact that human NOEL values do not necessarily describe the highest possible non-sensitizing concentration. However, the uncertainty in the correlation estimates was too high to conclude that either metric correlated significantly higher with cDV_0_ than the other. This is, however, a trend that could be considered in future works. 

Having shown that cDV_0_ values are associated with potency, a model capable of predicting NESILs was defined. Several approaches could be considered for creating prediction models that take cDV_0_ as input, including the creation of separate models for the classification of NESILs derived from LLNA EC3 and human NOELs (i.e., distinct models trained on LLNA EC3 and on human NOELs). However, this may not be ideal, as the same cDV_0_ value could give rise to two distinct potency values, which could impede interpretation. Therefore, it was explored if a single potency model could be defined from a composite potency reference constructed using information from both LLNA EC3 and human NOELs. A similar endeavor has been described in the construction of the reference chemical potency list (RCPL), where a consensus potency value was defined by evaluating both human and animal data [62]. While that scale, in part, relies on expert judgment for potency value assignment, the approach taken here was solely data- and computationally driven, combining NESILs from LLNA EC3 and human NOELs. The main assumption for the creation of the potency scale was that both LLNA EC3 and human NOELs describe the same underlying phenomenon, i.e., sensitizing potency, and that both metrics are associated with errors. The composite potency value was then designed to attempt to describe the common underlying potency signal, which was accomplished using a Passing–Bablok regression model [55] that robustly captured the association between the two references. Importantly, models fitted to the composite potency values received lower errors when predicting the original reference potency values, as compared with models fitted to the individual references. Moreover, the correlation coefficients were also marginally higher for the composite potency values compared with correlations to the individual references when considering the same set of 25 chemicals. While the reason for these results is not completely understood, it is possible that composite potency values inflict some degree of moderation or regularization to the original references, which may have a stabilizing effect during model fitting. This does not seem unreasonable, as a latent signal may be better described via the aggregation of several noisy measurements.

During initial model exploration, it was observed that the fitted parameters for the slopes were very close to 1, suggesting that they may not need to be estimated from the model. Indeed, models without an explicit estimate for the slope achieved lower values of Akaike’s Information Criterion (AIC) [63]. Because of this, for the actual evaluation of the models’ performances in cross-validation, they were fitted both with and without a parameter for the slope. In addition, the biological implication of a fixed slope of 1 could further indicate that the system used for the evaluation of potency is relevant, as the relative difference of skin-sensitizing potency is sustained. The consistently best-performing models corresponded to those without slope estimates. This is also convenient, as it leads to very simple prediction models. As the final models were fitted to log-transformed potency values and only contained estimates for the intercepts, in practice, they can be applied by multiplying an untransformed cDV_0_ value with a constant, as specified by equations 7 and 8. Moreover, as noted, an appealing property of these models is that they suggest that an n-factor difference in cDV_0_ values corresponds to an n-factor difference in the predicted NESIL. Finally, as models only contain a single estimated parameter, the uncertainties of the fits become relatively small and constant over the prediction range on the log scale. 

The errors for the prediction model were estimated at 2.75 and 3.22 fold changes compared with LLNA EC3 and human NOELs, respectively, evaluated using a cross-validation approach. These figures were also close to the errors observed for the fitted values of the final model (i.e., as predicted by the current fits without cross-validation), which were 2.69 fold changes and 3.12 fold changes for LLNA EC3 and human NOELs, respectively. In line with observations for the correlation coefficients, LLNA EC3 was predicted with a lower error, though not significantly. As previously noted, human NOEL does not necessarily represent the highest non-sensitizing concentration of a chemical, which could potentially explain prediction discrepancies. Despite this, the numbers of over/under-predicted chemicals were quite evenly balanced. Nevertheless, of the 12 chemicals whose potency was underpredicted compared with human NOELs, LOEL values could be located for 10. Interestingly, only three of the underpredicted potency instances produced predicted NESILs greater than the LOEL values (i.e., 7/10 predictions were between NOEL and LOEL).

To put the performance figures into greater context, the prediction performances of other potency sources were also considered. First, the error of the LLNA assay when predicting human NOELs in this dataset was calculated, and was found to be 2.63 fold changes. The error of the GARDskin Dose–Response model was 2.94 fold changes, compared with human NOELs on the same set of 25 chemicals. While the LLNA achieved an overall lower error, the prediction errors were not found to be significantly different. Other sources of potency predictions include, e.g., the relatively recent publication where several regression models incorporating data from established NAMs, including KeratinoSens, DPRA, h-CLAT, and the kDPRA, for predicting EC3 values were described. While their prediction metrices were based on a larger dataset (*n* = 188), they reported geometric mean absolute fold changes that ranged between 3.1 and 3.5 (median values ranged between 2.3 and 2.7). This appears to be comparable with the errors described in this work. One aspect of consideration is, however, that the prediction model proposed in this work is very simple and only requires a single information source, i.e., the cDV_0_ value, for potency predictions.

Considering the largest prediction errors obtained with this dataset, GARDskin Dose–Response predicted three chemicals with a fold change error greater than 5 compared with both human and LLNA references. These were alpha-isomethylionone, 2,4-dinitrochlorobenzene, and 3-propylidenephthalide. The potency of 3-propylidenephthalide was underpredicted by approximately 6-fold. It had two similar cDV_0_ values, suggesting that the assay outcomes were reproducible for the test item. Moreover, the NESILs from the LLNA and human NOEL were also very similar to each other. Currently, no explanation has been recognized for this prediction discrepancy. The potency of alpha-isomethylionone was overpredicted compared with both LLNA and human NOELs. The chemical had two runs in the GARDskin Dose–Response assay. However, these differed more than 4-fold, indicating some uncertainty in the chemical’s actual cDV_0_. Even so, a prediction generated with the larger of the two cDV_0_ estimates still overpredicted the potency of the chemical. The relative error for the run with the higher cDV_0_ value compared with LLNA EC3 was approximately 2.5 fold changes, which is, nevertheless, still a significant overprediction of potency compared with the human NOEL (>25 fold). It is relevant to note that the LLNA also overpredicts the potency of alpha-isomethylione. A tentative hypothesis for LLNA’s overprediction was previously provided where it was suggested that the autoxidation of the chemical, which is more likely to occur in the LLNA than under the occluded patches in HRIPTs, could explain the higher potency in the LLNA, as the oxidation products are typically strong sensitizers [64]. Although only a hypothesis, it is possible that this effect is also relevant for the overprediction of its potency in the GARDskin Dose–Response assay, which could potentially be explored in follow-up studies. Lastly, 2,4-dinitrochlorobenzene displayed a clear and steep response and the cDV_0_ value was considered reliable, despite only consisting of data from a single experiment. Interestingly, the underprediction of its potency does not appear to be a unique observation in the GARDskin Dose–Response assay, as similar tendencies were also described by Natsch et al. [65]. A clear explanation for this is, however, not available. Finally, it is important to consider that the GARDskin Dose–Response assay represents a relatively simple model of biological processes and ignores potential components that may contribute to the observed in vivo potency values. Additional data generated from chemical assessments in the GARDskin Dose–Response assay will likely provide interesting avenues for future investigations. These will help to further establish the predictive performance of the assay and other important properties, including the reproducibility of the numerical readout. Indeed, data for these purposes are being generated and some have already been published [41].

To conclude, we have described the GARDskin Dose–Response assay and demonstrated a significant association between its readout and available measures of skin-sensitizing potency on a set of 30 chemicals. A simple model for the prediction of sensitizing potency in the form of a quantitative continuous PoD was developed that only consisted of a single parameter. The performance of the prediction model was evaluated using cross-validation, and errors were estimated at 2.75 fold changes compared with NESILs derived from the LLNA, and 3.22 fold changes compared with NESILs based on human NOELs. The GARDskin Dose–Response method is therefore considered a useful tool for deriving skin-sensitizing potency information, which may be used as a PoD in subsequent risk assessments.

## Figures and Tables

**Table 1:** Description of the chemical dataset. The cPV column describes the computed composite potency values. Chemicals that have data from repeated tests show results from the individual studies within parentheses and the summarized values (the geometric mean), which are used for subsequent calculations, outside the parentheses. Entries with dashes (“-”) represent reference data points that could not be located or composite potency values that could not be defined due to at least one missing reference value.

				NESIL μg/cm^2^			
Chemical	CAS	MW (g/mol)	cDV0 (μg/mL)	LLNA	HRIPT NOEL	Composite Potency Value (cPV)	Human LOEL (μg/cm^2^)
Benzalkonium chloride	8001-54-5	424.15	0.350	25.0	-	-	-
2,4-Dinitrochlorobenzene	97-00-7	202.55	0.443	13.5	8.8	9.80	8.8
Cinnamic aldehyde	104-55-2	132.16	0.524	250	591	378	775
Citral	5392-40-5	152.23	1.11 (0.737, 1.67)	1450	1417	1440	3876
Diethyl maleate	141-05-9	172.18	1.03 (0.754, 1.40)	525	1600	921	-
Dimethyl fumarate	624-49-7	144.13	0.874	87.5	88	82.8	-
Methylisothiazolinone	2682-20-4	115.15	0.904	325	15	63.4	-
Benzyl Alcohol	100-51-6	108.14	1.37	NS	5905	-	8858
Chlorpromazine	50-53-3	318.9	1.38	35.0	1150	200	17,241
alpha-Isomethylionone	127-51-5	206.32	3.10 (1.48, 6.51)	5450	70,860	21,400	-
Iodopropynyl butylcarbamate	55406-53-6	281.09	1.61	225	-	-	-
Isoeugenol	97-54-1	164.21	1.70	325	250	275	775
p-Mentha-1,8-dien-7-al	2111-75-3	150.22	2.41 (1.73, 3.38)	1010	709	835	2760
Phenylacetaldehyde	122-78-1	120.15	2.68	750	591	654	1181
Carvone	6485-40-1	150.22	5.13 (3.58, 7.35)	3250	2657	2980	18,898
7-Hydroxycitronellal	107-75-5	172.26	5.70	5275	4960	5260	5814
5-Methyl-2,3-hexanedione	13706-86-0	128.169	8.97	6500	3448	4830	3450
Eugenol	97-53-0	164.21	9.29	2900	5906	4270	-
Ethyl acrylate	140-88-5	100.12	9.47	8188	1600	3630	4000
Cinnamic alcohol	104-54-1	134.17	10.3	5775	2953	4200	4724
Butyl resorcinol	18979-61-8	166.22	8.83 (10.3, 7.55)	950	-	-	-
Farnesol	4602-84-0	222.37	11.8 (11.5, 12.1)	1200	2755	1850	6897
Geraniol	106-24-1	154.25	15.4 (12.7, 18.7)	4025	11,811	7220	-
Imidazolidinyl urea	39236-46-9	388.29	14.9	6000	2000	3490	2000
3-Propylidenephthalide	17369-59-4	174.2	18.5 (18.8, 18.2)	925	945	928	2760
Pentachlorophenol	87-86-5	266.34	20.1	5000	2155	3310	6897
(R)-(+)-Limonene	5989-27-5	136.23	14.8 (20.2, 10.8)	13,125	10,000	12,000	-
3-Dimethylaminopropylamine	109-55-7	102.18	25.7	875	-	-	-
Benzyl salicylate	118-58-1	228.25	37.4	725	17,715	3790	-
Linalool	78-70-6	154.25	43.0	8875	14,998	12,100	-

**Table 2:** Linear (Pearson) and rank (Spearman) correlation coefficients between GARDskin Dose–Response cDV_0_, LLNA EC3, and human NOEL values.

	On Molar-Based Concentrations		On Mass-Based Concentrations	
	Linear Correlation	Rank Correlation	Linear Correlation	Rank Correlation
cDV_0_ vs. LLNA EC3	0.787 (*p* = 4.20 × 10^−7^)	0.709 (*p* = 2.74 × 10^−5^)	0.743 (*p* = 3.92 × 10^−6^)	0.669 (*p* = 7.18 × 10^−5^)
cDV_0_ vs. human NOEL	0.645 (*p* = 3.70 × 10^−4^)	0.664 (*p* = 3.08 × 10^−4^)	0.652 (*p* = 3.11 × 10^−4^)	0.656 (*p* = 2.74 × 10^−4^)
LLNA EC3 vs. human NOEL	0.738 (*p* = 2.54 × 10^−5^)	0.773 (*p* = 1.02 × 10^−5^)	0.736 (*p* = 2.72 × 10^−5^)	0.709 (*p* = 7.20 × 10^−5^)

**Table 3:** Regression coefficients of the PB models fitted between the reference NESILs. The values within the parentheses describe the estimated 95% confidence intervals of the coefficients.

	Intercept	Slope
Molar	0.0435 (−0.459, 0.331)	0.988 (0.700, 1.30)
Mass	0.183 (−0.778, 1.22)	0.958 (0.624, 1.22)

**Table 4:** Results from repeated cross-validation using individual references for fitting the models. The fold changes describe the absolute geometric mean fold changes from the cross-validation.

Potency Reference	Regression Method	Concentration Unit	Fold-Change Error—Intercept and Slope	Fold-Change Error—Intercept Only
LLNA	Linear regression	Mass	2.97	2.84
LLNA	Linear regression	Molar	2.96	2.84
NOEL	Linear regression	Mass	3.45	3.24
NOEL	Linear regression	Molar	3.45	3.24
LLNA	Robust regression	Mass	2.98	2.82
LLNA	Robust regression	Molar	2.98	2.82
NOEL	Robust regression	Mass	3.5	3.24
NOEL	Robust regression	Molar	3.48	3.24

**Table 5:** Coefficients of the fitted potency models. Values within the parentheses describe the estimated 95% confidence intervals for the coefficients.

Model	Estimated Intercept
Molar-based robust regression	−0.521 (−0.689, −0.353)
Mass-based robust regression	2.48 (2.32, 2.65)

## Data Availability

Data are contained within the article and Supplementary Materials.

## Supplementary Materials

The following supporting information can be downloaded at: https://www.mdpi.com/article/10.3390/toxics12090626/s1, Table S1: Chemical information; Tables S2 and S3: Dilution schemes for chemicals assayed in GARDskin Dose-Response.
