# Supplementary material for: In Vitro Prediction of Skin-Sensitizing Potency Using the GARDskin Dose–Response Assay: A Simple Regression Approach
Source: Toxics. 2024 Aug 24;12(9):626. doi: 10.3390/toxics12090626 (PMC11435491; doi:10.3390/toxics12090626)
Supplement: Supplementary file 1 [file toxics-12-00626-s001.zip › Supplementary Tables S2-S3.pdf]

Dilution schemes for chemicals assayed in GARDskin Dose-Response

Table S2. Chemicals from previously published studies (Gradin et al., 2021).

**Chemical**	**Concentration (µM)**
2,4-Dinitrochlorobenzene	8.3, 5, 3, 1.8, 1.1, 0.65, 0.39, 0.23, 0.14
3-Dimethylaminopropylamine	500, 300, 180, 108, 64.8, 38.9, 23.3, 14
7-Hydroxycitronellal	500, 300, 180, 108, 64.8, 38.9, 23.3, 14
Benzalkonium chloride	3, 1.8, 1.1, 0.65, 0.39, 0.23, 0.14, 0.084, 0.05
Benzyl alcohol	500, 300, 180, 108, 64.8, 38.9, 23.3, 14, 8.4, 5.04
Benzyl salicylate	334, 200, 120, 72.1, 43.2, 25.9, 15.6, 9.34, 5.6
Cinnamic aldehyde	60, 36, 21.6, 13, 7.8, 4.7, 2.8, 1.7, 1
Diethyl maleate	167, 100, 60, 36, 21.6, 13, 7.78, 4.67, 2.8
Dimethyl fumarate	90, 54, 32, 19, 12, 7, 4.1, 2.5, 1.5
Eugenol	500, 300, 180, 108, 64.8, 38.9, 23.3, 14, 8.4
Farnesol	108, 64.8, 38.9, 23.3, 14, 8.4, 5.04
Geraniol	500, 300, 180, 108, 64.8, 38.9, 23.3, 14
Imidazolidinyl urea	50, 30, 18, 10.8, 6.5, 3.9, 2.3, 1.4, 0.84
Iodopropynyl butylcarbamate	16.7, 10, 6, 3.6, 2.16, 1.3, 0.78, 0.47, 0.28
Isoeugenol	500, 300, 180, 108, 64.8, 38.9, 23.3, 14, 8.39, 5.04
Linalool	500, 300, 180, 108, 64.8, 38.9, 23.3, 14
Methylisothiazolinone	16.7, 10, 6, 3.6, 2.2, 1.3, 0.78, 0.47, 0.28
Pentachlorophenol	150, 90.1, 54, 32.4, 19.5, 11.7, 7, 4.2, 2.5

Gradin, R., Forreryd, A., Mattson, U., Jerre, A., & Johansson, H. (2021). Quantitative assessment of sensitizing potency using a dose–response adaptation of GARDskin. *Scientific Reports*, *11*(1), 18904. https://doi.org/10.1038/s41598-021-98247-7

Table S3. Chemicals acquired for this study.

**Chemical**	**Concentrations run 1 (µM)**	**Concentrations run 2 (µM)**
Citral	2 x (65.0, 32.5, 16.2, 8.12, 4.06, 2.03)	3 x (65.0, 39.0, 23.4, 14.0, 8.42, 5.05)
Diethyl maleate	2 x (200, 100, 50, 25, 12.5, 6.25)	
Chlorpromazine	2 x (10.0, 5.00, 2.50, 1.25, 0.625, 0.312)	
alpha-Isomethylionone	2 x (325, 162, 81.2, 40.6, 20.3, 10.2)	3 x (195, 117, 70.2, 42.1, 25.3, 15.2)
p-Mentha-1,8-dien-7-al	2 x (200, 100, 50.0, 25.0, 12.5, 6.25)	3 x (200, 120, 72.0, 43.2, 25.9, 15.6)
Phenylacetaldehyde	2 x (100, 50.0, 25.0, 12.5, 6.25, 3.12)	
Carvone	2 x (779, 390, 195, 97.4, 48.7, 24.3)	3 x (779, 467, 280, 168, 101, 60.6)
5-Methyl-2,3-hexanedione	2 x (70.0, 35.0, 17.5, 8.75, 4.38, 2.19)	
Ethyl acrylate	2 x (500, 250, 125, 62.5, 31.2, 15.6)	
Cinnamic alcohol	2 x (500, 250, 125, 62.5, 31.2, 15.6)	
Butyl resorcinol	2 x (200, 100, 50, 25, 12.5, 6.25)	3 x (200, 120, 72.0, 43.2, 25.9, 15.6)
Farnesol	2 x (150, 75.0, 37.5, 18.8, 9.38, 4.69)	
Geraniol	2 x (500, 250, 125, 62.5, 31.2, 15.6)	
3-Propylidenephthalide	2 x (405, 202, 101, 50.6, 25.3, 12.7)	3 x (405, 243, 146, 87.5, 52.5, 31.5)
(R)-(+)-Limonene	2 x (500, 250, 125, 62.5, 31.2, 15.6)	3 x (500, 300, 180, 108, 64.8, 38.9)
